# Imported zoonotic *Ancylostoma ceylanicum* and *Ancylostoma braziliense* infections in a cat in Romania

**DOI:** 10.1186/s13071-026-07429-7

**Published:** 2026-05-21

**Authors:** Ioana Bianca Mitrea, Mihai Sorin Cernea, Angela Monica Ionică, Andrada Silvia Cârstolovean, Sze Fui Hii, Patsy A. Zendejas-Heredia, Lucas Huggins, Vito Colella, Andrei Daniel Mihalca

**Affiliations:** 1 Department of Parasitology and Parasitic Diseases, Faculty of Veterinary Medicine, University of Agricultural Sciences and Veterinary Medicine of Cluj-Napoca, Calea Mănăștur 3‑5, 400372 Cluj-Napoca, Romania; 2 Department of Pharmacology, Faculty of Veterinary Medicine, University of Agricultural Sciences and Veterinary Medicine of Cluj-Napoca, Calea Mănăștur 3‑5, 400372 Cluj-Napoca, Romania; 3 Clinical Hospital of Infectious Diseases of Cluj-Napoca, Iuliu Moldovan 23, 400348 Cluj-Napoca, Romania; 4 Faculty of Science, University of Melbourne, Parkville, VIC 3052 Australia; 5 Parasitology Consultancy Group, 407056 Corușu, Romania

**Keywords:** Hookworms, *Ancylostoma ceylanicum*, *Ancylostoma braziliense*, Zoonosis, Romania, Molecular diagnosis

## Abstract

Hookworms are widespread zoonotic parasites affecting humans and companion animals worldwide. We report molecular characterization of *Ancylostoma ceylanicum* and *A. braziliense* in a cat imported into Romania from Guadeloupe, an overseas administrative region of the French Republic in the Caribbean. This finding underscores the role of molecular diagnostics in detecting the transboundary introduction of exotic hookworms with significant zoonotic potential.

**Graphical Abstract:**

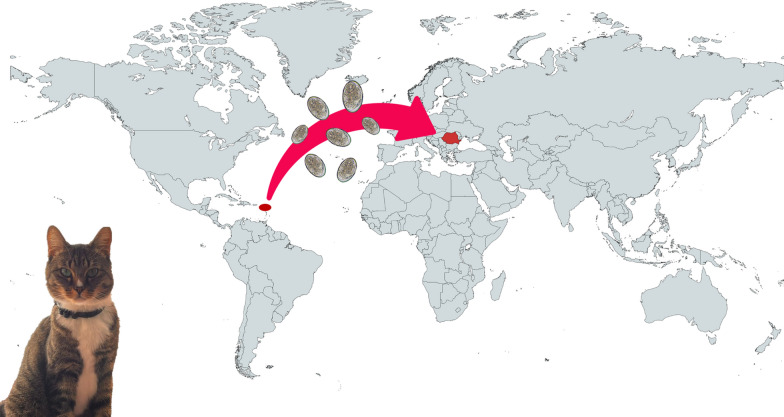

**Supplementary Information:**

The online version contains supplementary material available at 10.1186/s13071-026-07429-7.

Hookworms are hematophagous soil-transmitted helminths that parasitize the digestive tracts of mammals. Hookworm species infecting humans rank among the most common infections and affect millions of people in low- and middle-income countries [1].

*Ancylostoma caninum* is the predominant hookworm infecting wild and domestic canids, and species such as *Ancylostoma braziliense* and *Uncinaria stenocephala* occur in both canids and felids [2]. *Ancylostoma caninum*, *A. braziliense*, and *U. stenocephala* can penetrate human skin, producing cutaneous larva migrans that can cause intense itching and secondary bacterial infections [3].

Traditionally, only *. Ancylostoma duodenale* and *Necator americanus* were recognized as responsible for hookworm disease in humans. However, *A. ceylanicum*, is now recognized as the only animal hookworm capable of producing patent infections in humans [4]. It has emerged as an important zoonotic species infecting humans, particularly in Southeast Asia and the Pacific region [5], where dogs serve as reservoirs [2]. Reports of human and animal infections have also been documented in South and Central America, Africa, and Australia [6, 7]. To date, *A. ceylanicum* has not been reported in animals in Europe.

Here, we report a coinfection of *A. ceylanicum* and *A. braziliense* in a cat imported from Guadeloupe, an overseas department and region of the French Republic in the Caribbean, to Romania and discuss the potential risk of its establishment and the importance of molecular tools for accurate diagnosis.

A 3-year-old neutered female European shorthair cat (Hoshi) was presented to the Veterinary Clinic of the University of Agricultural Sciences and Veterinary Medicine (USAMV) Cluj-Napoca, Romania, on 24 March 2025 for frequent early morning urination. Clinical examination and behavioral observation indicated lower urinary tract discomfort. Hematology, biochemistry, urinalysis, and abdominal ultrasonography revealed no major abnormalities other than mild intestinal irregularities and metabolic acidosis, and the case was diagnosed as feline idiopathic cystitis.

The qualitative coprological examination with Sheather’s flotation (specific gravity 1.27) revealed the presence of eggs suggestive of hookworm infection. The fecal sample was then submitted for molecular analyses (see below). The cat was treated with Milprazon^®^ 16/40 mg (KRKA) (milbemycin oxime and praziquantel), according to the label instruction, and a subsequent fecal examination performed at 4 months using sugar flotation was negative. For cystitis, the cat received supportive care and management of clinical signs , along with dietary modification.

The history revealed that the cat was born in Belgium (3 June 2022) and lived there until August 2023. The cat moved with its owner to the French insular department of Guadeloupe, where it resided from August 2023 to December 2024. During this period, the cat lived indoors for the first month but was subsequently allowed to roam outside for the rest of the stay. Veterinary care was accessible, but visits were made only as needed. In December 2024, the owner and cat spent 2 weeks in continental France before settling in Cluj-Napoca, on 4 January 2025. The cat showed no clinical signs until its presentation to the clinic (March 2025).

The fecal sample was divided into two aliquots, one processed at USAMV Cluj-Napoca and the other at the University of Melbourne, Australia. In Cluj-Napoca, genomic DNA was isolated from the feces using the DNeasy PowerSoil Pro Kit (QIAGEN, Germany) according to the manufacturer’s protocol and then molecularly screened by internal transcribed spacer 2 (ITS-2) polymerase chain reaction (PCR) [8]. The obtained sequences were assembled and edited using Geneious^®^ software 2026.0.1 (Biomatters LTD., Auckland, New Zealand) and compared with existing sequences from GenBank^®^ by means of basic local alignment search tool (BLAST) analysis. At the University of Melbourne, DNA was extracted from feces using a modified Maxwell^®^ RSC PureFood GMO and Authentication Kit protocol on the Maxwell^®^ RSC 48 Instrument (Promega, USA) [9], followed by two multiplex quantitative (q)PCR [10], targeting the ITS-1 rRNA region of *A. caninum*, *A. braziliense*, *A. ceylanicum*, and *U. stenocephala*. For further cross-validation and to obtain a more informative hookworm species barcoding region from this sample, a novel parasitic nematode metabarcoding method on the Oxford Nanopore Technologies platform was employed [11].

The sequenced conventional PCR product (232 bp) identified *A. ceylanicum* (100% nucleotide identity and 100% query cover) with more than 20 other *A. ceylanicum* isolates, e.g., JF960369, JN164659, HQ452520, and JQ673419. The sequence was deposited in GenBank under the accession number PX867001. The multiplex qPCR confirmed *A. ceylanicum*, with a Cq value of 23.88 and *A. braziliense* with a Cq value of 24.62. Nanopore metabarcoding of the parasitic nematode ITS1-5.8S-ITS2 barcoding region generated a total of 114,307 reads. After quality filtering, consensus clustering and classification, a total of 71,588 reads were classified as *A. ceylanicum* (100% nucleotide identity and 93% query cover to accession PP527745 across 771 bp) and 30,560 reads were classified as *A. braziliense* (99% nucleotide identity and 99% query cover to accession JQ812692 across 784 bp). Nanopore consensus sequences were added to the National Center for Biotechnology Information (NCBI)’s GenBank under accession numbers PX927951 and PX927952.

Sequences from *A. braziliense* and *A. ceylanicum* were used for phylogenetic reconstruction (Fig. 1). ITS region sequences were retrieved from GenBank and aligned in Geneious Prime, yielding a final alignment of 220 bp. The best-fit nucleotide substitution model was identified in MEGA v12, and phylogenetic relationships were inferred using both Bayesian inference and maximum likelihood approaches. Haplotypes were identified from 304 sequences representing *A. ceylanicum*, *A. braziliense*, *A. tubaeforme*, and *A. caninum* using DNAcollapser (Supplementary Table 1).

**Figure 1:**
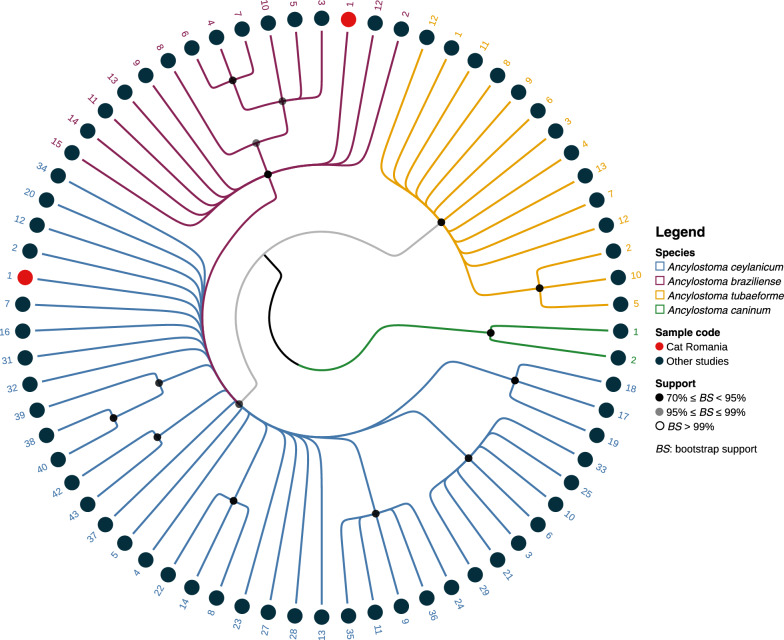
Phylogenetic analysis was conducted using Bayesian inference in MrBayes v3.2.7a, applying the Jukes–Cantor (JC) substitution model with four gamma-distributed rate categories. Four independent Markov chains were run for 1,000,000 Markov chain Monte Carlo (MCMC) generations, with trees sampled every 200 generations. The first 25% of sampled trees were discarded as burn-in, and the remaining trees were used to construct a majority-rule consensus tree. Convergence was assessed by ensuring that the potential scale reduction factor approached 1.0 and that the average standard deviation of split frequencies was < 0.01. Maximum-likelihood (ML) analysis was performed to support the Bayesian tree using 5000 bootstrap replicates on the basis of Akaike information criterion (AIC). TreeViewer v2.20 was used to annotate the ML tree. Numbers above the circles indicate haplotype IDs. Node support is indicated by circles representing ML bootstrap support (BS) values. Haplotype details, including GenBank accession numbers and country of origin, are provided in Supplementary Table 1

Here, we document the occurrence of *A. ceylanicum* and *A. braziliense* in Europe. The global movement of companion animals and people increases the risk of introducing exotic hookworm species into previously unaffected regions, raising concerns about their potential establishment and onward transmission in nonendemic settings. This infected cat most likely represents an imported case, with the infection probably originating from the Caribbean, a region where *A. ceylanicum* and *A. braziliense* occur in canids [12, 13]. However, to our knowledge, no data are available for Guadeloupe.

Although the endemicity of *A. ceylanicum* in Europe cannot be excluded, its environmental suitability would likely be seasonal. Larval development depends on temperature and humidity, with optimal intra-egg development at 20–30 °C [14] and progression to the infective stage favored by moist, sandy, organic-rich soils, conditions present in parts of Romania [15].

Warmer months in regions such as Cluj-Napoca [15] may enable larval survival and seasonal transmission, provided infected animals contaminate the environment and susceptible hosts are exposed to the infective larval stages. However, the temperate climate is unlikely to support year-round development. Consequently, long-term establishment would depend on sufficient numbers of infected definitive hosts, allowing nematode populations to persist through winter. Coprological examination is the standard method for detecting hookworm eggs, but it has low sensitivity and hookworm species cannot be differentiated morphologically [16]. Consequently, routine fecal screening may miss uncommon or exotic species, particularly in imported animals or travelers from endemic areas. Molecular approaches, including PCR and TaqMan-based multiplex qPCR assays, have been effectively used to detect mixed natural infections in fecal samples and to estimate the effectiveness of control strategies [17]. More recently, the development of a long-read metabarcoding approach capable of detecting all parasitic nematodes simultaneously has been effectively used to identify rare or unexpected species that might otherwise be missed by conventional molecular approaches [11].

Current European legislation regulating the movement of companion animals (e.g., EU pet travel scheme) primarily focuses on rabies prevention and identification, and in specific cases, the control of certain zoonotic parasites such as *Echinococcus multilocularis.* However, it may not sufficiently address the risk of introducing exotic parasitic infections, such as certain *Ancylostoma* spp. Moreover, movements between overseas territories and mainland EU countries, such as from Guadeloupe to continental France, may be subject to less strict controls than animals imported from non-EU territories, increasing the risk of parasite introduction.

This report highlights the introduction of exotic hookworm species into Europe through the movement of companion animals and emphasizes the importance of molecular diagnostics for their accurate and timely detection and monitoring.

## Supplementary Information

Supplementary material 1: Table 1. Sequences from hookworms used for phylogenetic construction.

## Data Availability

All data generated or analyzed are provided within the manuscript and in the Supplementary information files.
