# Supplementary material for: Imported zoonotic Ancylostoma ceylanicum and Ancylostoma braziliense infections in a cat in Romania
Source: Parasit Vectors. 2026 May 21;19:252. doi: 10.1186/s13071-026-07429-7 (PMC13281336; doi:10.1186/s13071-026-07429-7)
Supplement: Supplementary file 1 — Supplementary material 1: Table 1. Sequences from hookworms used for phylogenetic construction. [file 13071_2026_7429_MOESM1_ESM.docx]

Supplementary Table 1. Sequences from hookworms used for phylogenetic construction

| **Species** | **Haplotype** | **GenBank accession number** |
| --- | --- | --- |
| *Ancylostoma braziliense* | 1 | **Romania Cat (Guadeloupe)**  MG890384 (Tanzania)  PP930863, PP930875 (Tanzania)  JQ812693 (USA)  PX590272 (Cambodia)  MK203855 (Australia) |
|  | 2 | MK203855 (Australia) |
|  | 3 | MG890379 (Tanzania) |
|  | 4 | MG890386 (Tanzania) |
|  | 5 | MG890388 (Tanzania) |
|  | 6 | MG890383 (Tanzania) |
|  | 7 | MG890382 (Tanzania) |
|  | 8 | MG890387 (Tanzania) |
|  | 9 | DQ359149, DQ438051, OR801626-27, OR804050, PQ326408-15 (India)  PP93087, PP930859-62, PP930864-72, PP930874, PP930876-80 (Malaysia) |
|  | 10 | MG890385 (Tanzania) |
|  | 11 | JQ812692 (USA) |
|  | 12 | DQ438050, DQ438052-53, DQ438055-58, DQ438060-64, DQ438066-69 (Brazil) |
|  | 13 | DQ438054 (Brazil) |
|  | 14 | DQ438065 (Brazil) |
|  | 15 | DQ438059 (Brazil) |
| *Ancylostoma ceylanicum* | 1 | **Romania-Cat (Guadeloupe)**  DQ381541, DQ780009, OP715867, PP527745 (India)  KF279132, KF279134, KF279136 (China)  LC036567 (Papua New Guinea)  OR826944-51 (Indonesia)  PP798007, PP798008-42, PP798044-61, PP798063-91, PP798095-96, PP798098-100, PP798102-PP798114 (Malaysia)  PX590270 (Cambodia) |
|  | 2 | AB501355 (Laos)  KX577784-85 (Thailand) |
|  | 3 | DQ831517 (Australia)  KF279138 (China) |
|  | 4 | DQ831518 (Australia) |
|  | 5 | DQ831519 (Australia) |
|  | 6 | DQ831520 (Australia) |
|  | 7 | JX317642, JX840460-63, KY640230, KY676846, KY681815, MG733994, MG904962, MG904964 (China) |
|  | 8 | KC755015 (China) |
|  | 9 | KC755020 (China) |
|  | 10 | KC755021 (China) |
|  | 11 | KC755027, KF279137 (China)  PP798062, PP798092 (Malaysia) |
|  | 12 | KC896798 (China) |
|  | 13 | KF279133 (China) |
|  | 14 | KF279135 (China) |
|  | 15 | KM066110 (Germany, migrant from Colombia) |
|  | 16 | KU996382, KU996386 (India) |
|  | 17 | KU996383, KU996387 (India) |
|  | 18 | KU996384, KU996385 (India) |
|  | 19 | LC177188, LC177190-91, LC177193, LC177196-99, LC177202-203 (Laos) |
|  | 20 | LC177189, LC177206 (Laos) |
|  | 21 | LC177200 (Laos) |
|  | 22 | MG589493 (China) |
|  | 23 | MG719974 (China) |
|  | 24 | MG719975 (China) |
|  | 25 | MG719977 (China) |
|  | 26 | MG890212 (China) |
|  | 27 | MG890213 (China) |
|  | 28 | OL454900 (Viet Nam) |
|  | 29 | ON773142 (Ecuador) |
|  | 30 | ON773143 (Ecuador) |
|  | 31 | OR342818, OR342820-24 (Thailand) |
|  | 32 | OR342819 (Thailand) |
|  | 33 | PP798021 (Malaysia) |
|  | 34 | PP798043 (Malaysia) |
|  | 35 | PP798093 (Malaysia) |
|  | 36 | PP798094 (Malaysia) |
|  | 37 | PP798097 (Malaysia) |
|  | 38 | PX497935 (Laos) |
|  | 39 | PX497936 (Laos) |
| *Ancylostoma tubaeforme* | 1 | JQ812691-4 (USA) |
|  | 2 | KY474053 (China) |
|  | 3 | KY474054 (China) |
|  | 4 | KY474055 (China) |
|  | 5 | MG589509 (China) |
|  | 6 | MG589510 (China) |
|  | 7 | MG589514, MG904955, MG904957-60, (China) |
|  | 8 | MG589516 (China) |
|  | 9 | MG589517, MG865903 (China) |
|  | 10 | MG589519, MG733992, MG904956 (China) |
|  | 11 | MG589636 (China) |
|  | 12 | PP812123 (Germany) |
|  | 13 | PQ316556-57 (Iran)  PX557812 (Romania) |
| *Ancylostoma caninum* | 1 | JQ812694 (USA) |
|  | 2 | JQ812694 (USA) |
